# Enthalpic Classification of Water Molecules in Target–Ligand Binding

**DOI:** 10.1021/acs.jcim.4c00794

**Published:** 2024-08-13

**Authors:** Viktor Szél, Balázs Zoltán Zsidó, Csaba Hetényi

**Affiliations:** Pharmacoinformatics Unit, Department of Pharmacology and Pharmacotherapy, Medical School, University of Pécs, Szigeti út 12, Pécs 7624, Hungary

## Abstract

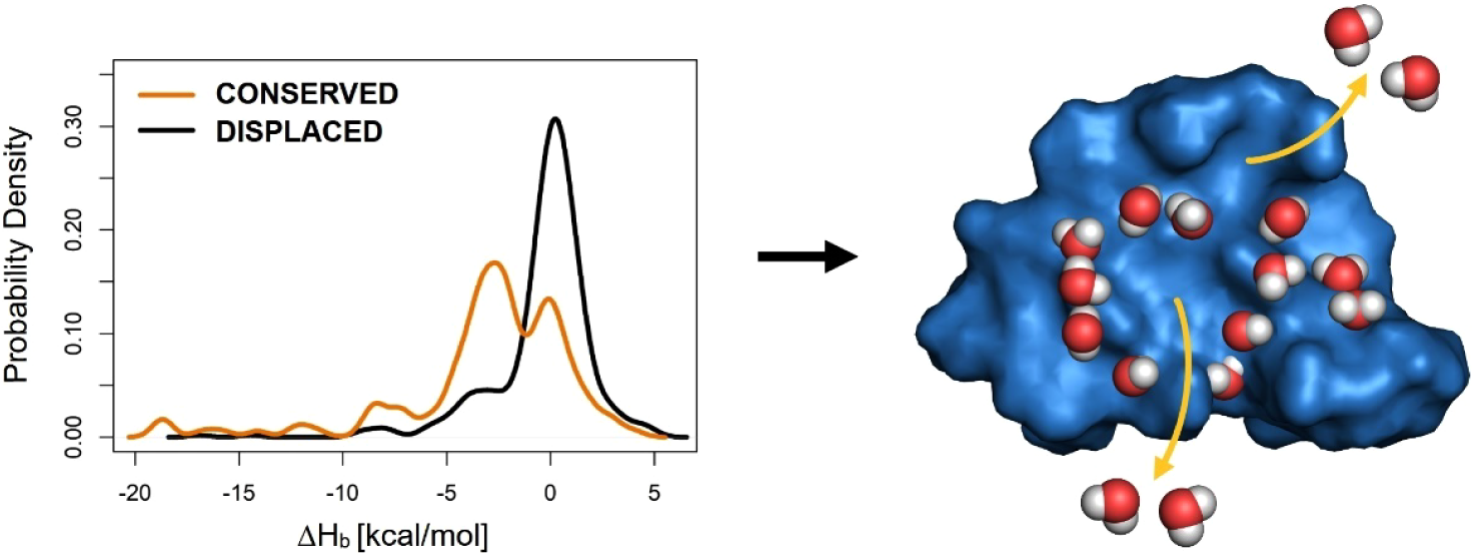

Water molecules play various roles in target–ligand binding. For example, they can be replaced by the ligand and leave the surface of the binding pocket or stay conserved in the interface and form bridges with the target. While experimental techniques supply target–ligand complex structures at an increasing rate, they often have limitations in the measurement of a detailed water structure. Moreover, measurements of binding thermodynamics cannot distinguish between the different roles of individual water molecules. However, such a distinction and classification of the role of individual water molecules would be key to their application in drug design at atomic resolution. In this study, we investigate a quantitative approach for the description of the role of water molecules during ligand binding. Starting from complete hydration structures of the free and ligand-bound target molecules, binding enthalpy scores are calculated for each water molecule using quantum mechanical calculations. A statistical evaluation showed that the scores can distinguish between conserved and displaced classes of water molecules. The classification system was calibrated and tested on more than 1000 individual water positions. The practical tests of the enthalpic classification included important cases of antiviral drug research on HIV-1 protease inhibitors and the Influenza A ion channel. The methodology of classification is based on open source program packages, Gromacs, Mopac, and MobyWat, freely available to the scientific community.

## Introduction

**Figure 1:**
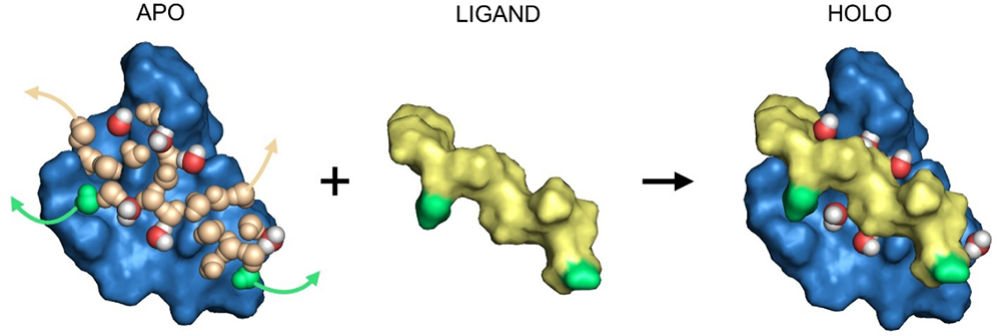
A schematic representation of the conserved and displaced water positions. The scheme is based on the binding of the peptide ligand Ac-APTMPPPLPP-NH_2_ to the protein target tyrosine kinase on the hydration structure (System 1abo). The target pocket and ligand molecule are displayed in marine blue and light yellow surfaces, respectively. Water positions (spheres) of the apo target surface (left) can be classified as either conserved (red/white or green) or displaced (wheat). Water molecules that physically leave (arrows, left) the target surface join the bulk during ligand binding. Thus, the conserved water positions include: (1) physical water molecules stay in place and form bridges (or fill space) between target and ligand (right, red/white spheres), and (2) positions of water molecules that are isosterically replaced (green arrows) by proper ligand groups (right, green ligand surface). For clarity, not all water molecules are shown. The figure was prepared using PyMol.^30^

Elucidation of the role of individual water molecules in target–ligand binding is essential^1^ for precise drug design and biomolecular engineering.^2,3^ The different roles of water structure were demonstrated on important drug targets, including HIV-1 protease,^4^ TYK2 kinase,^5^ JAK3 kinase,^5^ PDE4^6^ enzyme targets, adenosine A2A receptor^7^, or filling human or viral^8−12^ ion channels. However, the exploration of the role of individual water molecules is often hindered by the limitations^13,14^ of experimental techniques that can determine water positions at atomic resolution. Experimental structure determination techniques often cannot produce a complete hydration structure with all the water molecules bound in the first hydration shell of the molecular surface of a target.^13,15,16^ This problem has been partially^17^ overcome by the development of theoretical approaches,^1,13,14^ and their combinations with experiments.^18^ However, there are only a few methods^19−24^ supplying the water structure of the unbound (apo) target surface as a starting point (Figure 1) of the target-ligand complexation. On the other end, a number of theoretical methods^12,16,21−23,25−29^ can produce the water positions, the target–ligand interface (the end-point in Figure 1) of the holo form at a high precision.

**Figure 2:**
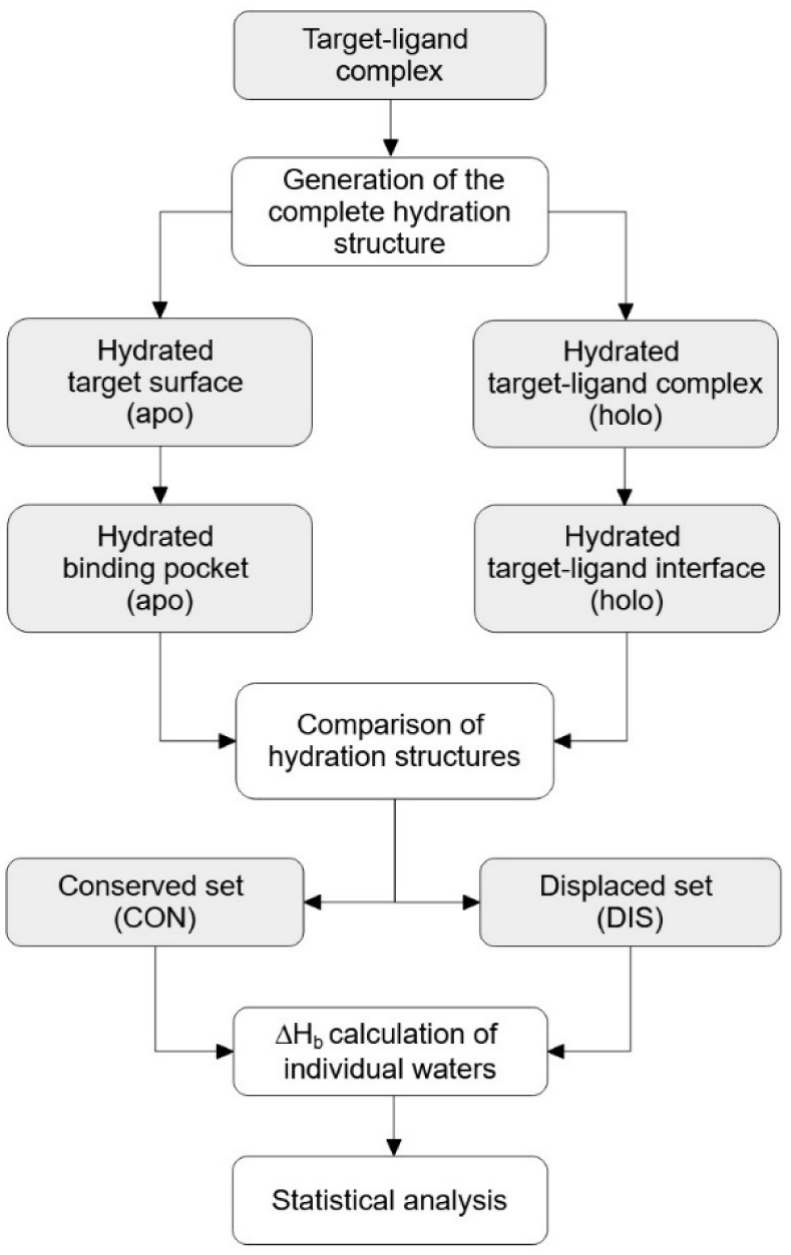
Main steps of the investigation of enthalpic classification of water positions.

Water positions are usually classified into conserved (staying, happy) and displaced (leaving, unhappy) groups. In the present study, conserved water positions include: (1) physical water molecules that stay in place and form bridges (or fill space) between target and ligand (red/white spheres, on the right of Figure 1), and (2) positions of water molecules that are isosterically replaced (green arrows) by proper ligand groups (right, green ligand surface). The groups of conserved and displaced water positions can be generated from the apo surface and holo interface water structures (Figure 1), as the subtraction of the set of holo interface plus the isosterically replaced waters from the set of all the apo surface waters yields the set of displaced waters. The two groups have radically different roles in drug design. For example, conserved waters usually have an important role in mediating target–ligand interactions and, therefore, they can be included in docking/screening studies^1^ as part of the target. They can be also used as a template for a water-mimicking group with binding features similar to (better than) the template water during lead optimization.^31−33^ Displaced water molecules contribute to the desolvation thermodynamics of ligand binding.

There have been structural (Table S1) and thermodynamic (Table S2) approaches introduced for the above classification of water positions. In the former group, there are knowledge-based approaches^24,34−40^ that align protein structures available in the Protein Databank,^41,42^ involving clustering techniques, and resulting in consensus hydration sites with probability scores. There are fewer methods that perform the above-mentioned systematic comparison of the hydration structures of apo–holo protein pairs applying various models of *K*-nearest neighbors,^38^ logistic regression,^39^ ranking,^40^ and structural descriptors like B-factor^39^ (Table S1). They usually show good performance on the selected training/validation sets for discrimination between conserved and displaced waters. However, such knowledge-based methods work on previously determined water positions, and are limited by the available crystallographic data used for their training.

Thermodynamic methods^22,23,28,43^ provide an alternative and use first-principles for classification of water molecules according to their energetic contribution to target–ligand or target–water interactions (Table S2). For example, the comparison of target–ligand complex sets and an alchemical double decoupling approach resulted^43^ in a systematic differentiation between conserved and displaced water sets for ligand design. They found that the binding free energy contributions of individual water molecules (Δ*G*_b_) were more negative (favorable) in the conserved set on average than in the displaceable set, which allowed a classification between the two sets for ligand design. Other methods estimate both enthalpic and entropic binding contributions and use molecular dynamics (MD) sampling to generate an ensemble of structures with individual water property distributions and utilize clustering to generate hydration sites.^22,28^ WATsite^23,44^ incorporates waters with favorable (Δ*G*_b_ < 0) contribution into a pharmacophore model to assist screening on multiple targets. WaterMap^22^ is another MD-based method, where inhomogeneous fluid solvation theory provides Δ*G*_b_ for individual hydration sites. The above-mentioned thermodynamic approaches for classification of water molecules work in the molecular mechanics framework without explicit quantum mechanical (QM) calculations of electronic effects and polarization. However, the large dipole moment of water often induces such effects in the surrounding partners necessitating the involvement of QM calculations, which was shown to improve the calculation of target–ligand interactions.^45^ It was further demonstrated that single-point semiempirical QM methods accurately calculate Δ*G*_b_^46,47^ and enthalpy change (Δ*H*_b_)^46^ of ligand binding. State-of-the-art QM-based approaches of drug design were recently summarized in excellent reviews.^48,49^

The present study investigates whether the classification of conserved and displaced water molecules is possible based on their binding enthalpy changes (Δ*H*_b_) to the apo target surface, that is, without any information on the bound structure of the ligand molecule. A QM-based calculation of Δ*H*_b_ is introduced to account for all the changes in the interactions, including the above-mentioned electronic effects. The assessment of the classification is based on the statistical comparison of complete hydration structures generated for various target–ligand systems.

## Methods

### Systems

Target–ligand complex structures (Table S3) were obtained from the Protein Databank. For the apo water prediction and classification procedure, the input target structures were from the respective target–ligand (holo) pdb structures in the case of 1abo, 1hcs, 1lcj, 2qbx, 2rod, and 2jm6 systems. For the HIV-protease target apo structure, 1g6l was used as a consensus structure for the comparison of 11 different bound ligands. Crystallographic waters and other solvent molecules and ions were removed from the files. Missing solute atoms were built with Swiss PDB Viewer.^50^ Ligands of systems 1abo, 1hcs, and 1lcj were equipped with acetyl and amido groups using PyMol^30^ according to our previous study.^46^ In the cases of systems 1hcs and 1lcj the force field parameters of the phosphotyrosine (pY) residue was adopted from our previous study.^51^

### Generation of Hydration Structures

Water positions of the unliganded (apo) target binding pocket and the target–ligand (holo) interface for all systems were determined by MobyWat^19,25^ based on MD runs. According to the MobyWat surface and interface hydration protocols, the procedures were first applied on the dry target and subsequently on the target–ligand complex, respectively. The detailed method was presented earlier,^19^ shortly described here in the following sections, remarking the applied differences.

#### Molecular Dynamics

Prior to the MD run, the solute (the apo target or the target–ligand complex) was energy-minimized at the molecular mechanics level in a two-step fashion, including steepest descent (sd) and conjugate gradient (cg) algorithms. The hydrogenated solute was placed in a cubic box by using a distance criterion of 1 nm between the solute and the edge of the container. The box was filled up with explicit TIP3P water molecules, and counterions (sodium or chloride) were added to neutralize the system. Exit tolerance levels were set to 10^3^ and 10 kJ·mol^–1^·nm^–1^, while maximum step sizes were set to 0.5 and 0.05 nm for the sd and cg steps, respectively. Position restraints were applied to solute heavy atoms at a force constant of 10^3^ kJ·mol^–1^·nm^–2^. Calculations were performed with programs of the GROMACS software package,^52^ using the AMBER99SB-ILDN force field.^53^

After energy-minimization, 1 ns-long NPT MD simulation was carried out with a time step of 2 fs. For temperature-coupling, the velocity rescale and the Parrinello–Rahman algorithms^54−56^ were used. Solute and solvent were coupled separately, with a reference temperature of 300 K and a coupling time constant of 0.1 ps. Pressure was coupled by the Parrinello–Rahman algorithms and a coupling time constant of 0.5 ps, compressibility of 4.5 × 10^–5^ bar^–1^ and reference pressure of 1 bar. Particle mesh–Ewald summation was used for long-range electrostatics. van der Waals and Coulomb interactions had a cutoff at 11 Å. Coordinates were saved at regular time-intervals of 1 ps yielding 1 × 10^3^ frames. Position restraints were applied to solute heavy atoms at a force constant of 10^3^ kJ·mol^–1^·nm^–2^. Periodic boundary conditions were treated before analysis to make the solute whole and recover hydrated solute structures centered in the box. Each frame was fit to the original protein crystal structure by using Cα atoms. The final trajectories, including all atomic coordinates of all frames, was converted to portable binary files.

#### Calculation of Water Positions on Apo (Unliganded) Target and the Holo (Ligand-Bound) Target–Ligand Complex

From the apo MD simulation data surface water positions were calculated with the all-inclusive identity-based (IDa) prediction algorithm of MobyWat.^19,25^ Maximum distance from target (dmax), prediction, and clustering tolerances were set to 5, 2.5, and 1 Å, respectively. First, candidate water molecules for all frames were selected based on a desired distance limit (dmax) from the target, and then an occupancy list was constructed containing every different water ID on every line and the respective number of occurrences as candidates among all frames. Clustering was applied to all rows (all different water IDs) of the occupancy list by using a clustering tolerance (ctol) parameter to define the distance between the elements of the same cluster. The largest cluster was selected from all to give the first predicted water oxygen atom by averaging the spatial coordinates of the included molecules. In the further steps, clusters were selected with decreasing size and checked if their distance was larger than the prediction tolerance (ptol) from previously predicted water oxygen positions.

The target and the predicted water oxygen atoms were merged into one file, hydrogens were added to the system, and energy minimization was performed according to a four-step protocol sd-cg-sd-cg pattern with parameters of sd and cg methods described previously. During the first two steps, all solute heavy atoms and the oxygen of the predicted interface water molecules were position restrained, and bulk waters and ions were released. In the last two steps, position restraints were not applied on predicted waters, only on solute heavy atoms. The protocol resulted in an optimized and hydrated apo target structure for all systems (Table S3 and Figure 2).

In the cases of the holo systems, the target, the ligand, and the water oxygen atoms predicted for the apo target were merged, and water oxygens clashing with the ligand were excluded using the Editing mode of MobyWat. Following the MD run, water structure was predicted from the holo trajectories, and the four-step minimization was performed with the same settings as was described previously in the apo case. The optimized and hydrated holo target–ligand complex structure was piped into the next steps of the investigation (Table S3 and Figure 2).

#### Extraction of Hydrated Binding Interfaces and Pockets

Target–ligand binding interfaces were cut out from the hydrated holo structures with the Fragmenter server^51^ after prediction and minimization steps of the previous sections. Target–ligand and solute–water cutoff distances were set to 8 and 5 Å, respectively, and the extracted target chain fragments were equipped with acetyl groups (N-terminals) and methylamino groups (C-terminals). Binding pockets of the hydrated apo target structures were also extracted using the same parameters. In this case, the experimental ligand structure of the respective holo complex was included in the apo files to define the surrounding target residues of the holo interface based on the distance from the ligand. In some cases, cutting parameters had to be adjusted to obtain exactly the same residues for both apo and holo structures, despite the slight movements during MM minimization. The fragmentation procedure was repeated for all the systems and resulted in the structures of extracted hydrated target binding pockets and target–ligand interfaces of all systems. The apo and holo coordinates are available in PDB format (see Data Availability).

### Formation of Sets of Conserved and Displaced Water Positions

Pair-wise comparison of the unliganded binding pocket (apo) and target–ligand interface (holo) structures was performed for each system to sort the water positions of the binding pocket into two sets. A water position in the unliganded binding pocket was considered conserved and placed into the CON_*x*_ set (CON_*x*_, where *x* refers to the system code in Table S3), if either (1) a matching water position was found in the target-ligand interface or (2) a matching, isosteric heteroatom (O or N) position was found in the ligand molecule. Water–water matching (case 1) was performed at a match tolerance (mtol, the distance between the oxygen atoms in the surface and interface water positions) of 1.0 Å. On the other hand, water–ligand matching (case 2) was done at an mtol (distance between the surface water oxygen atom and ligand heteroatom) of 1.75 Å.

The displaced sets (DIS_*x*_) of water positions were separated from all the apo water positions in the unliganded binding pocket excluding those already selected for the conserved set (Figures 1 and 2). The final counts of conserved and displaced water molecules are listed in Table S4. Data files on the structural classification results are available (see Data Availability).

### Calculation of Binding Enthalpy Values of Individual Water Molecules

All bound water molecules were removed from the extracted binding pocket of the target and inserted back one by one to calculate the water–target binding enthalpy (Δ*H*_b_) for individual water molecules according to eq 1. For this, single point (1SCF) calculations were performed by MOPAC2016^57^ at the semiempirical level using PM7^58^ parametrization, COSMO implicit solvation model,^59^ and MOZYME localized molecular orbital approach.^60^ Keywords of PM7 1SCF EPS = 78.3 MOZYME were specified. The 1SCF calculations were performed, including only the extracted binding pocket of the target and one water molecule at a time, and resulted in formation enthalpy (Δ_f_*H*) values of the Target:H_2_O complex (eq 1). Formation enthalpies of the dry binding pocket of the target (Target in eq 1) and a free water molecule (H_2_O in eq 1) were also calculated by using the same 1SCF procedure. Then, the binding enthalpy (Δ*H*_b_) of a specific water to the target was calculated from the Δ_f_*H* values according to Hess’s law (eq 1). The whole procedure was repeated for all water molecules present in the binding pocket, and the corresponding series of Δ*H*_b_ values were stored for statistical analyses (Figure 2). The calculated Δ_f_*H* and Δ*H*_b_ values are available in data files (see Data Availability).
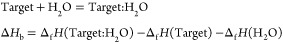
1

### QM Minimization of Water Positions

To check the effect of QM minimization on Δ*H*_b_, the water positions of previously MM-minimized and fragmented systems were QM-minimized with MOPAC2016^57^ at the PM7^58^ level with MOZYME^60^ linear scaling and COSMO^59^ implicit solvation (EPS = 78.3). Position restraint was applied on target heavy atoms, while all hydrogens and water oxygens were minimized at a gradient norm exit threshold of 3 kcal/mol (GNORM = 3). Using the QM minimized structures, individual water Δ*H*_b_ values were calculated as presented before.

### Case Studies

For the systems of the case studies (Table S3), hydrated apo target (binding pocket) structures were produced as it was described in the Generation of hydration structures.

#### Identification of HIV-1 Protease Inhibitors with an Oxygen Atom Isosteric to the Predicted Conserved Water Molecules

Water positions were generated on the surface of the binding pocket of the apo HIV-1 protease enzyme structure (PDB 1g6l) as described before. The apo enzyme was used, as it has an unbiased (unliganded) geometry and hydration structure for comparison with the ligand-bound (holo) complexes. The HIV-1 protease holo enzymes in complex with inhibitors (Table S5) were superimposed onto the MobyWat-hydrated apo enzyme by Cα atoms using PyMol^30^ align function. The corresponding Cα RMSD values were smaller than 1 Å and are listed in Table S5. The distance between the oxygen atom of the water molecule classified as conserved (on the apo enzyme surface) and the closest oxygen atom of the enzyme-bound inhibitor molecule (*d*_W–I_) was used to select those ligands which have an oxygen atom isosteric to the conserved water molecule. The *d*_W–I_ values were measured using PyMol for all conserved waters and inhibitors and listed in Table 1 (nonminimized water positions were used). An inhibitor was identified to have an oxygen atom isosteric to the conserved water if the *d*_W–I_ < 1.75 Å (in this case, the conserved water served as a template during inhibitor design).

**Table 1:** Distances Between the Oxygen Atom of a Conserved Water Position and the Closest Oxygen Atom of the Inhibitor Molecules (*d*_W–I_, Å) of 11 HIV-1 Protease-Inhibitor Complexes

	conserved water/Δ*H*_b_ (kcal/mol)		
inhibitor	W1^a^/–14.41	W10/–4.26	W2/–9.34
Amprenavir	2.2^b^	**0.9**	2.7
Atazanavir	**0.8**	2.4	2.8
Darunavir	2.2	**0.9**	2.5
DMP323	**1.1**	**0.7**	**0.7**
Indinavir	2.0	**1.1**	2.9
Lopinavir	**1.7**	**1.3**	1.9
Nelfinavir	**0.9**	2.2	2.8
Ritonavir	**1.5**	**1.6**	2.3
Saquinavir	**1.2**	1.9	2.9
Tipranavir	**1.3**	2.2	**0.8**
U89360E	**1.1**	2.3	2.9
Count of inhibitors with a conserved water used as isosteric template	8	6	2

a The serial number of the water molecule with the lowest numbers corresponds to the best Δ*H*_b_. W1 and W10 are at residue D25, and W2 is at residue I50 of the HIV-1 protease, respectively.

b If the distance (*d*_W–I_, Å) of the oxygen atom of a conserved water molecule from a the closest oxygen atom of an inhibitor is below 1.75 Å (bold) the water molecule was identified as an isosteric template (its position was used in the inhibitor during the original design).

#### Identification of Displaceable Water Molecules in the Design of Influenza A Ion Channel Inhibitors

Water positions were generated on the inner surface of the Influenza A ion channel after the removal of amantadine from the complex (PDB code 6bkk). The Influenza A virus ion channel in complex with spiro-adamantyl amine (PDB code 6bmz) was superimposed onto the hydrated nonminimized holo enzyme 6bkk by Cα atoms using PyMol^30^ align function. The corresponding Cα RMSD value was 0.4 Å. The *d*_W–I_ values were measured using PyMol for all waters and both inhibitors as described before; the difference was that not only ligand oxygen atoms were considered for the measurement but also all ligand heavy atoms. A displaceable water molecule was identified if the *d*_W–I_ < 1.75 Å.

### Statistics

All statistical calculations and plotting was done with the program R.^61^ For the histograms multiple bar widths were tried out, and 1 kcal/mol was found to be adequate. Kernel density estimation technique was used for fitting estimated probability density curves on the Δ*H*_b_ data with a bandwidth parameter of 0.5 kcal/mol. Sensitivity is defined as the value of the fitted empirical cumulative density function (CDF), while selectivity is the ratio of two CDFs as a function of Δ*H*_b_. For the boxplots lower whisker, lower quartile (25th percentile), median (50th percentile), upper quartile (75th percentile), and upper whisker markers were considered. The lower whisker is defined as the smallest value greater than the lower quartile minus the 1.5 times the interquartile range (IQR), while the upper whisker as the largest value lower than the upper quartile minus 1.5 times the IQR.

Receiver operating curves (ROC) were fitted and analyzed using R^61^ packages ROCR and cutpointr. The efficiency of the classification was evaluated with the area under the curve (AUC) metric of the ROC curves. Optimal classification thresholds were determined with the F1-score metric given by eqs 2**–**4, where *P* is positive, *N* is negative, *TP* is true positive, *PP* is predicted positive, and *TN* is true negative value. At the optimal classification thresholds, accuracy values were also calculated (eq 5)
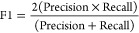
2
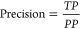
3
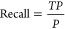
4
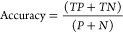
5

### Water Interaction Networks

Hydration networks were generated from the optimized hydration structure of the apo binding pockets of the systems 1lcj and 2jm6 using MobyWat’s NetDraw^25^ mode. Maximum heavy atom distance (dmax) of 3.5 Å and maximum mobility limit (mmax) of 50 was used as input parameters. Output files (.gdf) contain a list of nodes and edges of a graph representation of the H-bonding network of the systems. A node is classified as static if it is either a solute (target or ligand) node, connected to four nodes of any type, or connected to at least three solute nodes. In every other case, the node is dynamic. An edge is static if it connects two static nodes; otherwise, it is dynamic. For visualization of the graphs, Gephi open-source network analysis software package^62^ was used with Force Atlas rendering mode augmented with attraction distribution and adjust size settings.

## Results and Discussion

### Enthalpic Classification

In the present study, important targets, including tyrosine kinase, receptor tyrosine kinase (2qbx), and leukemia differentiation protein were chosen that are essential in signal transduction pathways (Table S3). These proteins bind large peptide ligands, resulting in extensive binding interfaces that supply several water positions for the present investigations of Δ*H*_b_-based classification. The work (Figure 2) started with the generation of conserved and displaced water molecules for all systems. Experimental techniques cannot guarantee the determination of complete water structures (Introduction), while previous studies^19,25^ demonstrated that an MD-based method, MobyWat, can generate water positions at high success rates. Thus, in the present study, MobyWat was applied to produce a void-free water layer on the surface of the unliganded binding pocket (apo form),^19^ and in the ligand-bound interface (holo form),^25^ respectively (Figure 2, Methods). Predicted water positions were also validated using available experimental (crystallographic) water positions of the investigated systems (Table S3) and a mean success rate of 90.2% was observed (Table S6). Finally, 687 and 446 individual water positions were obtained (Table S3) for the apo and holo forms, respectively, and used in the following steps of classification and calibration.

In the next step, pairwise comparisons of the water structures of the apo and holo forms resulted in the sets of conserved (CON_*x*_) and displaced (DIS_*x*_) water positions (Figure 2) for each system (*x*) of Table S3. The details of the formation of the sets are provided in the Methods, and the counts of water molecules in each set are listed in Table S4. The Δ*H*_b_ values of 687 individual water molecules to their respective apo targets were calculated according to Hess’s law (eq 1) with 1SCF calculations at the semiempirical quantum mechanical level. The corresponding lists of Δ*H*_b_ values were collected separately for the CON_*x*_ and DIS_*x*_ sets of each system of Table S3. The applied approximations (fragmentation of structures and single-point approach) did not show a significant effect on the calculated Δ*H*_b_ values (detailed results are provided for system 1lcj in Figures S1 and S2).

**Figure 3:**
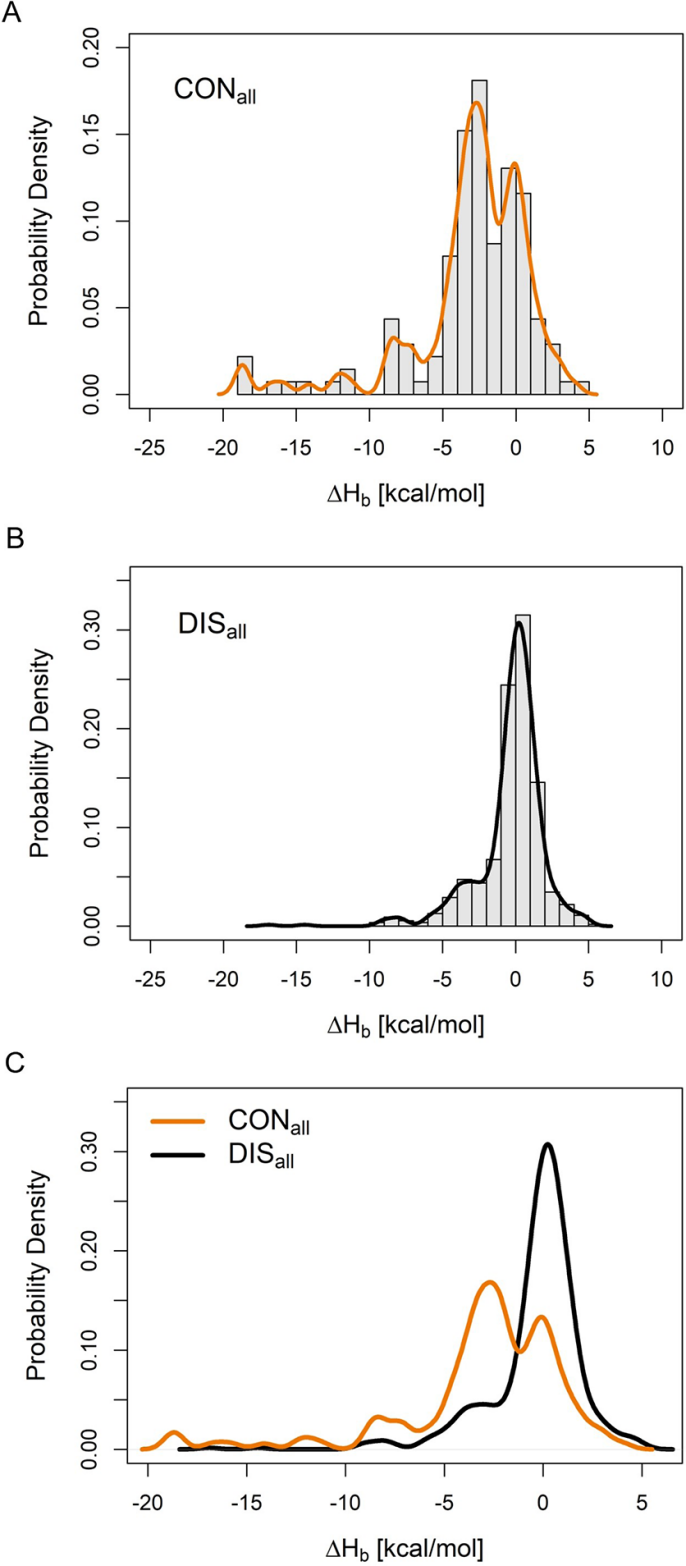
Histograms and the corresponding kernel density estimate curves (bandwidth = 0.5) of the CON_all_ (A) and DIS_all_ (B) water sets (mtol = 1 Å) that contain all water molecules of all systems. The kernel density estimate curves (C) are also displayed together for comparison.

In the general statistical evaluations, the apo water positions (and the corresponding Δ*H*_b_ values) of all systems of Table S3 were merged into two sets containing all conserved (CON_all_) and displaced (DIS_all_) sets, respectively (The “all” sets are the union of the above “x” sets). The descriptive statistics resulted in three probability density maxima observed on the histograms of both sets (Figure 3A,B) at Δ*H*_b_= 0.0, −3.0, and −8.0 kcal/mol, among which the highest peak corresponds to 0.0 kcal/mol, indicating that most of the water positions do not have a direct interaction with the target in both groups. Such waters are either located at apolar surfaces or in upper (mostly the second)^63^ hydration shells. The other two peaks at −3.0 and −8.0 kcal/mol can be assigned roughly to one and two water-target hydrogen bonds, respectively.

Probability density curves were also fitted with the kernel density estimation technique for an easier comparison of the two sets. The comparison of the curves shows that the distribution of the two sets overlap (Figure 3C), that is, the density peaks of the two sets are located approximately on the top of each other. At the same time, the DIS_all_ set has a probability density peak at 0.0 kcal/mol that is more than twice as high as that of the CON_all_ set (Figure 3C). On the other hand, the CON_all_ set has 3.5 and 4 times higher peaks than the DIS_all_ set at −3.0 and −8.0 kcal/mol, respectively.

These findings show that conserved waters can be distinguished from displaced waters by their Δ*H*_b_ values. Waters strongly interacting with the target (Δ*H*_b_ < −10 kcal/mol) are also represented by considerable density almost exclusively for CON_all_. These waters usually have 2 or more hydrogen bonds with charged side-chains (R,K,D,E) of the target. (A separate set of calculations were performed, where the neighboring waters of a given water were also included in the Δ*H*_b_ calculation and resulted in completely overlapping CON_all_ and DIS_all_ distribution curves showing no practical differences in local densities. Details of the calculations are presented in Figure S3).

**Figure 4:**
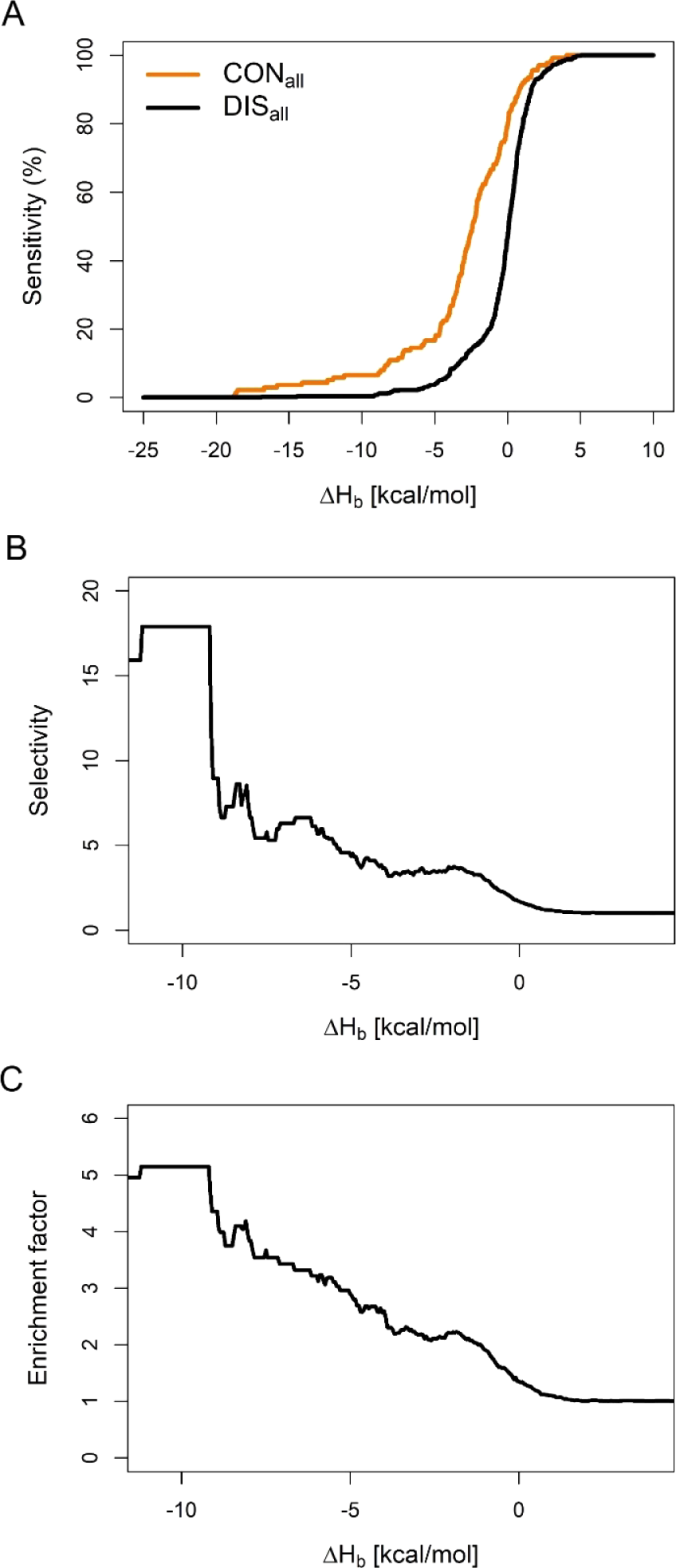
(A) From the cumulative probability density functions (100 × CDF, in %), the sensitivity of Δ*H*_b_ was obtained for the CON_all_ and DIS_all_ water groups (mtol = 1 Å), respectively. (B) The ratio of the CDFs of the two sets (CON_all_/DIS_all_) measures the selectivity of the Δ*H*_b_-based discrimination between the two groups in favor of conserved waters. (C) The enrichment factor (EF) can be also used as an alternative measure of selectivity at a given Δ*H*_b_ value.

As the overlapping Δ*H*_b_ distributions of CON_all_ and DIS_all_ sets have the above-mentioned considerable difference in their probability densities in the ca. Δ*H*_b_ < −3 kcal/mol domain, the selectivity of Δ*H*_b_ can be expressed as a measure of the distinction between conserved and displaced water molecules. Such selectivity of the Δ*H*_b_-based classification at a given Δ*H*_b_ value can be obtained from the ratio (Figure 4B) of the cumulative density functions (CDF, Figure 4A) obtained for the CON_all_ and DIS_all_ sets, respectively. The value of CDF alone at a given Δ*H*_b_ reports the sensitivity of the classification at a selected Δ*H*_b_ (Figure 4A). While the sensitivity is rather low (below 8%) in the Δ*H*_b_ < −10 kcal/mol region, such water molecules can be practically considered as conserved ones. Toward the more positive Δ*H*_b_ values, the selectivity decreases gradually with an increasing sensitivity (Figure 4A,B).

An ROC analysis was also applied to evaluate the efficiency of classification of conserved and displaced water sets. On the full data set, an overall 0.74 AUC value was obtained (Figure S4B) that further justified the ability of Δ*H*_b_ to distinguish between CON_all_ and DIS_all_ sets. Using the maximum of the F1-score metric, a default classification threshold of −1.9 kcal/mol can be obtained with an accuracy of 78.7% (Table S7). The selected threshold also shows a favorable selectivity of 3.7 and a sensitivity of 60% (Figure 4). Notably, if a higher selectivity of classification is required by the actual drug design project (that is, fewer waters with higher probability of conservation), then a threshold of −4.0 kcal/mol (or lower) can be applied, that value corresponds to a break-point of the selectivity function in Figure 4B. (The effect of QM minimization of water molecules on classification was also investigated in terms of ROC AUC values, and the results are provided in Table S8. The QM minimization of water molecules provided no improvement over 1SCF calculations in the distinction of conserved and displaced sets).

As another approach for measurement of the selectivity of Δ*H*_b_-based classification between conserved and displaced water molecules, the enrichment factor (EF) was also calculated. EF is a familiar term in virtual screening of drugs, and in our context, it measures the proportion of conserved waters among the top ranked (100α %) waters based on their Δ*H*_b_ compared to the same proportion in the entire set of waters. A higher EF indicates a better performance of the Δ*H*_b_-based selection of conserved waters.
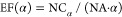
6

In eq 6, α is the top portion of the ranked waters, NC is the number of conserved waters in the top α portion, and NA is the number of all ranked waters. Similarly to the selectivity trend in Figure 3B, EF also decreases with increasing (more positive) Δ*H*_b_ (Figure 4C). For example, the above-mentioned F1-score-based Δ*H*_b_ threshold of −1.9 kcal/mol has a selectivity of 2.2 for conserved water molecules in terms of EF.

### System-Dependency of the Classification

**Figure 5:**
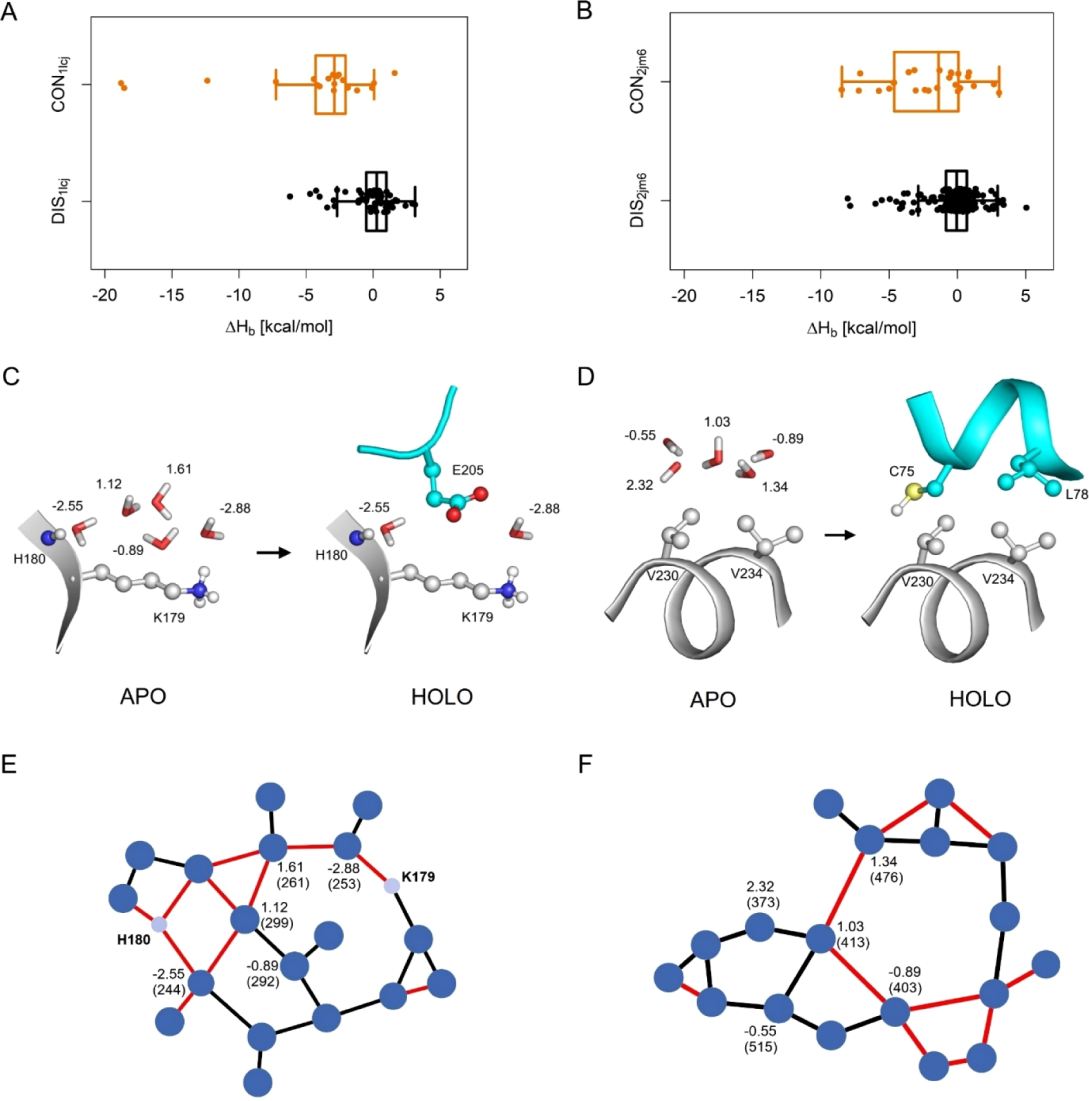
Classification of conserved (CON_*x*_) and displaced (DIS_*x*_) water sets of selected systems (*x*) with different polarities of the target binding pockets. (A, B) Descriptive statistics of the Δ*H*_b_ distributions displayed as boxplots and strip charts. Vertical lines of a boxplot represent the lower whisker, lower quartile (25th percentile), median, upper quartile (75th percentile), and upper whisker from left to right, respectively. The absolute value of the difference between the upper and lower quartiles is considered as the interquartile range (IQR). The lower whisker is the smallest value greater than the lower quartile minus the 1.5 times the IQR, while the upper whisker is the largest value lower than the upper quartile plus 1.5 times the IQR. (C, D) Examples of ligand binding to binding pocket residues of different polarity. Target residues are displayed in gray ball–stick/cartoon, ligand residues in cyan ball–stick/cartoon, and water molecules in stick representation. Δ*H*_b_ values are shown next to water molecules in kcal/mol units. In the unliganded (apo) case of 1lcj the charged ammonium group of K179 and backbone amido (NH) group of H180 target residues form hydrogen bonds with waters 244 and 253, respectively, reflected in their considerable negative binding enthalpies (C, **left**). Upon ligand binding (holo form), waters 261, 292, and 299 with unfavorable (or slightly favorable) binding enthalpy are displaced by ligand E205, while enthalpic waters form new hydrogen bonds with the carboxylate group, and this way they mediate the interaction between target and ligand (C, **right**). On the other hand water molecules have no contact with the V230 and V234 residues in the mostly nonpolar 2jm6 pocket (D, **left**). These waters form hydrogen bonds only with each other and have unfavorable (or slightly favorable) Δ*H*_b_ with the target leading to easy displacement by the ligand (D, **right**). (E, F) Local water interaction networks of the hydrated binding pockets of different polarity. (E) Residues K179 and H180 (gray filled circles) of system 1lcj anchor the hydrogen bonding network of water molecules of the binding pocket to the target (blue filled circles). K179 forms 1 static (red line) and 1 dynamic (black line), while K180 forms 3 static edges in the network graph. (F) The extensive H-bonding network can be also noticed in the 2jm6 pocket, but the neighboring target residues (V230 and V234) cannot anchor it to the target. Δ*H*_b_ values are shown next to water circles in kcal/mol units. Below the Δ*H*_b_ values, respective PDB residue serial numbers are also shown in parentheses. The network graphs were generated with MobyWat’s NetDraw mode^25^ and Gephi^62^ graph visualizer software.

Following the general assessment of the Δ*H*_b_-based classification of conserved and displaced water positions, an in-depth analysis was performed individually for all systems of Table S3. For this, CON_*x*_ and DIS_*x*_ sets were considered, where *x* denotes the system codes listed in Table S3. The results of the descriptive statistics are presented for two particular systems 1lcj and 2jm6 in Figure 5.The centers of CON_*x*_ sets are shifted toward the negative Δ*H*_b_ values for both systems (Figure 5A), when compared with the centers of DIS_*x*_ sets, although system 1lcj shows a higher degree of separation. The DIS_*x*_ sets are pretty similar and are centered around 0 kcal/mol in both cases (Figure 5 A,B). The interquartile ranges of the CON_*x*_ and DIS_*x*_ sets show no overlap for system 1lcj, while in the case of system 2jm6 a pronounced overlap was observed (Figure 5 A,B). The ROC analysis revealed similar results, where a more favorable classification efficiency was obtained on system 1lcj (AUC = 0.85) compared with 2jm6 (AUC = 0.67) (Figure S4A).

To interpret the above differences in the separability of conserved and displaced water positions in different systems, the polarity of the binding pockets were investigated by calculating their fractional polar surface area (FPSA, Å^2^). Details of the calculations are presented in Table S9. In Figure 5, two systems 1lcj and 2jm6 of different amino acid compositions of their binding pockets are featured. As it was mentioned above, Δ*H*_b_ provided the most effective differentiation between conserved and displaced waters in case of 1lcj (Figure 5A) with no overlap in interquartile ranges. In 1lcj, the binding pocket is composed of mostly polar (FPSA = 0.55, Table S9) and/or charged (R,K) groups extensively hydrated with water molecules of negative Δ*H*_b_ values interacting with e.g., target residues K179 and H180 (Figure 5C) that act as anchor points for the local water network (Figure 5E) of the binding pocket. During ligand binding (arrow in Figure 5C), waters of unfavorable, less negative (or even positive) Δ*H*_b_ values are displaced by the side-chain of residue E205 of the ligand, while the most negative ones remains conserved and form a H-bridge (yellow in Figure 5C) with the carboxylate of E205. Notably, the displaced waters of unfavorable Δ*H*_b_ were located around the (nonpolar) methylene groups of K179 before ligand binding.

In the counterexample of system 2jm6, a moderate separation (Figure 5B) was observed, which can be attributed to the nonpolar residues (FPSA = 0.29) dominating the binding interface (Table S9 and Figure 5D). In 2jm6, several V and F residues form a hydrophobic environment, and the water molecules tend to interact with each-other (Figure 5D) instead of the target resulting in more positive Δ*H*_b_ values. Thus, the local water network above, e.g., V230 and V234 has no anchor points on the target side (Figure 5F) and can be easily displaced during ligand binding (arrow in Figure 5D). A similar situation with a cage-like water network can be observed around other nonpolar residues like F299 (Figure S3).

The above examples showed that the distinction capability of Δ*H*_b_ between conserved and displaced water positions is vital for binding pockets of high polarity. This is reasonable since at polar, charged surfaces water molecules tend to spend most of the time at well-defined hydration sites, forming strong, specific hydrogen bonding interactions (large negative Δ*H*_b_) with suitable target groups. Such stable hydration sites also establish the opportunity for favorable bridging interactions during ligand binding (Figure 5C), and conservation of the water positions is essential for the design of target–ligand interactions. On the other hand, at nonpolar surfaces, in the absence of strong, directed H-bonding interactions with the target, waters with more positive Δ*H*_b_ values tend to interact with each other in networks of cage-like structures^64−67^ around nonpolar side chains and easily displaced by the ligand (Figures 5D and S5). Other water positions at nonpolar sites have just a peripheral role, and they remain conserved with a close to zero (or even positive, Figure 5B) Δ*H*_b_ indicating their marginal role in ligand design.

### Case Studies

**Figure 6:**
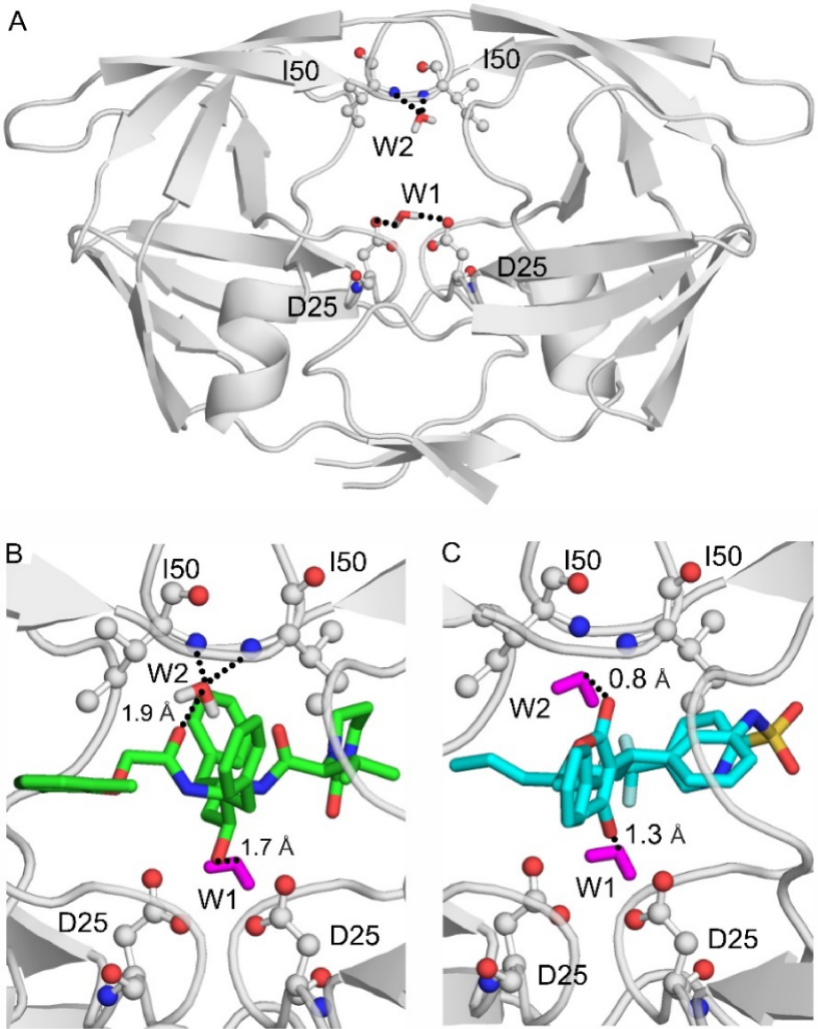
Conserved water molecules identified in the present study were used as templates in the design of HIV-1 protease inhibitors lopinavir and tipranavir. (A) Conserved water molecules W1 and W2 with the top Δ*H*_b_ values in CON_1g6l_ interact with D25 and I50 amino acids of the apo HIV-1 protease, respectively. HIV-1 protease is shown as gray cartoon, D25 and I50 amino acids are shown as balls and sticks, and water molecules as red and white sticks. (B) The closest oxygen atom of lopinavir (green sticks) and the oxygen atom of water W1 are isosteric (*d*_W–I_ < 1.75 Å, black dotted line). During the design of lopinavir W1 was used as a template to ensure the interactions of lopinavir with the two D25 amino acids. As water W1 is plausibly not present simultaneously with lopinavir in the binding site, it is colored to purple. W2 bridges between lopinavir and the I50 amino acids. (C) The closest oxygen atom of tipranavir (teal sticks) and the oxygen atoms of waters W1 and W2 are isosteric (*d*_W–I_ < 1.75 Å, black dotted line). During the design of tipranavir W1 and W2 were used as templates to ensure interactions of lopinavir with the two D25 and I50 amino acids. As waters W1 and W2 are plausibly not present simultaneously with tipranavir in the binding site, they are colored to purple. Hydrogen bonds of conserved water molecules with the target are represented as black dotted lines.

The previous sections showed that Δ*H*_b_ can be used to classify water molecules according to their roles in ligand binding. In the next sections, the applicability of the Δ*H*_b_ scores will be further tested in antiviral drug design situations. The first case study is focused on the design of HIV-1 protease inhibitors^68^ where water molecules were shown^68−79^ to have an influential role in drug action. The positions of water molecules of the first hydration layer were predicted for the binding pocket of the apo HIV-1 protease target (PDB code 1g6l) using the same method as described in the previous sections. Δ*H*_b_ values of the predicted water molecules were calculated for each individual water molecule. Using the threshold calibrated for the selection of conserved water positions in Enthalpic Classification, waters with top Δ*H*_b_ (<−1.9 kcal/mol) were selected for the CON_1g6l_ set (Table S10). To check the applicability of this classification for the HIV-1 protease system, it was investigated whether the shortlisted water molecules in set CON_1g6l_ have a significant structural role in the HIV-1-inhibitor complexes. Notably, the serial numbers of water molecules reflect their ranks in the list, where a smaller serial number corresponds to a more negative Δ*H*_b_. It was found that water molecules W1, W10, and W2 of the top Δ*H*_b_ values in CON_1g6l_ are interacting (Figure 6A) with the D25 and I50 amino acids of HIV-1, respectively (due to the C_2v_ symmetry of the homodimeric protease structure, both W1 and W10 were predicted to interact with the two neighboring D25 residues). Both D25 and I50 are important^69−79^ in binding inhibitors to the active centrum of the protease.

In the next step, it was investigated whether the shortlisted water positions were involved in previous inhibitor design. Namely, the atomic position of a water oxygen can be used as a template for a water-mimicking group of a protease inhibitor with similar (or better) binding features than the template water.^4^ That is, a new oxygen atom designed in the inhibitor is isosteric to the template water oxygen and directly interacts with the target forming a stronger H-bond with the target when compared to the water used as template.^1,32^ In addition, during ligand binding, the template water is replaced by the new, isosteric water oxygen yielding a favorable increase in entropy.^80^ To check if the shortlisted water positions can be selected as templates, spatial comparisons of the water positions and the structures of available inhibitor–HIV-1 protease complexes were performed. The distances between the oxygen atom of a shortlisted water position and the closest oxygen atom of the inhibitor molecules (*d*_W–I_) were measured and listed in Table 1. A water was identified as a template if *d*_W–I_ < 1.75 Å (close contact, Methods, Figure 6B,C). It was found that position W1 or W10 were used^70−79,81^ in all HIV-1 protease inhibitors, and W2 was used in tipranavir and DMP323 (Table 1). The corresponding original publications^70−79,81^ also verify the usage of these water positions. Thus, the above results show that the Δ*H*_b_-based classification managed to select conserved, top water positions that have been proven useful as templates in inhibitor design for HIV-1 protease.

**Figure 7:**
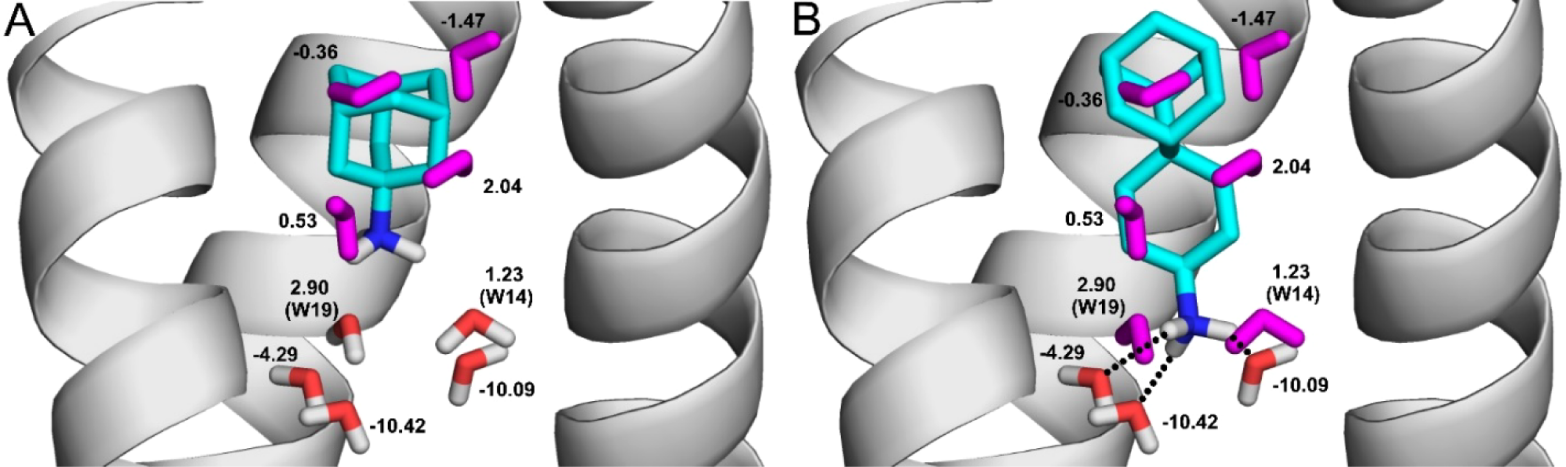
Role of water molecules covering the Influenza A virus channel. Influenza A virus ion channel is shown as gray cartoon, amantadine (A) and spiro-adamantyl-amine (B) are shown as teal sticks, and bridging water molecules as red and white thick lines. Displaced water molecules are colored in purple (not all water molecules are shown for clarity). Δ*H*_b_ values are shown for all water molecules in kcal/mol. Waters W14 and W19 displaced by spiro-adamantylamine (B), but not by amantadine (A) are also marked with their serial numbers in brackets. Hydrogen bonds between conserved water molecules and the ligand (B) are represented as black dotted lines.

In the second case study, the role of displaced water molecules with Δ*H*_b_ > −1.9 kcal/mol were investigated on the binding of ligands amantadine and spiro-adamantyl-amine to the Influenza A virus ion channel (PDB codes 6bkk, 6bmz, respectively). A list (Table S11) of water molecules and the corresponding Δ*H*_b_ values were generated as it was described in the previous sections. In this case, we investigated whether water molecules with Δ*H*_b_ > −1.9 kcal/mol sorted into the displaced DIS_6bkk_ set are indeed displaced during ligand binding or not. Following a similar procedure as in the first case study, the measurement of the *d*_W–I_ distances (here with the closest heavy atom on the ligand side) showed that 8 water molecules (Figure 7A) of the DIS_6bkk_ set with Δ*H*_b_ > −1.9 kcal/mol have *d*_W–I_ < 1.75 Å with amantadine, and therefore, they are indeed displaced during ligand binding. The larger ligand, spiro-adamantyl-amine also displaces an additional 3 water molecules (altogether 11 water molecules). Two of the additional 3 displaced water molecules W14 and W19 (Figure 7B) have positive Δ*H*_b_ values, and after their displacement, the protonated amino group of spiro-adamantyl amine can directly interact with conserved bridging water molecules placed at the top of the CON_6bkk_ list (red and white in Figure 7B, bold in Table S11). In this way, spiro-adamantyl-amine (Figure 7B) can be considered as a structurally improved ligand over amantadine (Figure 7A), that was reflected by a significant improvement of its IC_50_ value against a drug-resistant mutant^82^ as well.

All-in-all, the above case studies showed how conserved and displaced water molecules classified by their Δ*H*_b_ can be used in antiviral drug design.

## Conclusions

The present study investigated the use of calculated Δ*H*_b_ values for the classification of the role of individual water molecules during target–ligand interactions. The approach was statistically verified using more than 1000 water positions of relevant protein–ligand complexes, which is a strong test set considering the number of water positions used for verification of other methods (Tables S1 and S2). It was shown that Δ*H*_b_ can be applied as a score for classification of conserved and displaced water positions. The calculation of Δ*H*_b_ is based on a fast, end-point approach at the semiempirical QM level. The present study is unique in the sense that it applies a QM-based score for the classification of individual water molecules instead of an MM-based calculation of the thermodynamics of available methods (Tables S1 and S2). QM-based approaches have an obvious advantage of accounting for the electronic effects in intermolecular interactions, that may be particularly important in the case of the water molecule of a large dipole moment and the often highly charged protein surrounding. Accordingly, the development of QM-based approaches is an emerging trend in current drug design.^47−49^ The present study provides a tool that is compatible with other QM-based methods^47−49^ and useful in all cases where water molecules affect target–ligand interactions. Such cases of antiviral drug design were featured to show how the classified water molecules are selected as a template for building isosteric functional groups in HIV-1 protease inhibitors or displaced by Influenza A virus ion channel blockers. The methodology of classification is based on open source program packages, Gromacs, Mopac, and MobyWat, freely available for the scientific community.

## Data Availability

Compressed files, including predicted apo and holo structural (.pdb) files, MOPAC input (.mop), and output (.out) files; calculated Δ_f_H and Δ*H*_b_ values (.csv); and structural classification results of waters (.csv) are available at https://zenodo.org/records/13150315.

## Supporting Information Available

Methods for structural and thermodynamical characterization of waters; investigated systems; ROC curves; water structure at nonpolar surfaces; count of water molecules; HIV test set; ROC analysis results; fractional polar surface area of binding pockets; list of HIV-1 waters; list of influenza A waters (PDF)

## Notes

GROMACS (https://www.gromacs.org/.), MobyWat (http://mobywat.com/.), MOPAC (http://openmopac.net/.), PyMol (https://pymol.org/.), Swiss PDB Viewer (https://spdbv.unil.ch/.), R (https://www.r-project.org/.), and Gephi (https://gephi.github.io/) are all open-source softwares.
