# Supplementary material for: Enthalpic Classification of Water Molecules in Target–Ligand Binding
Source: J Chem Inf Model. 2024 Aug 13;64(16):6583–95. doi: 10.1021/acs.jcim.4c00794 (PMC11351019; doi:10.1021/acs.jcim.4c00794)
Supplement: Supplementary file 1 — ci4c00794_si_001.pdf [file ci4c00794_si_001.pdf]

Supporting Information

Enthalpic classification of water molecules in target-ligand binding

Viktor Szél, Balázs Zoltán Zsidó and Csaba Hetényi*

*Pharmacoinformatics Unit, Department of Pharmacology and Pharmacotherapy, Medical School, University of Pécs, Szigeti út 12, 7624 Pécs, Hungary. *Corresponding author. E-mail: hetenyi.csaba@pte.hu*

Table of Contents

Figure S1 The effect of fragmentation on calculated ΔH_b values 2
Figure S2 The effect of QM minimization on calculated ΔH_b values 2
Figure S3 The effect of neighbouring water molecules on the ΔH_b calculation 3
Figure S4 ROC analysis results and binding site polarity 4
Figure S5 Water structure in the proximity of non-polar target surface 5
Table S1 Methods for structural classification of binding site water molecules 6
Table S2 Methods for thermodynamic characterization of binding site water molecules 7
Table S3 Target-ligand systems investigated in the present study 8
Table S4 Counts of water molecules in different sets 9
Table S5 HIV test set experimental data collection 9
Table S6 Validation of predicted water positions 10
Table S7 Optimal classification thresholds and respective accuracy values 10
Table S8 ROC AUC values obtained for single point QM and QM minimization approaches 10
Table S9 Fractional Polar Surface Area of the binding pockets of investigated systems 11
Table S10 List of water molecules generated for the binding pocket of the HIV-1 protease 12
Table S11 List of water molecules generated for the binding pocket of the influenza A ion channel 13
References 14

Figures

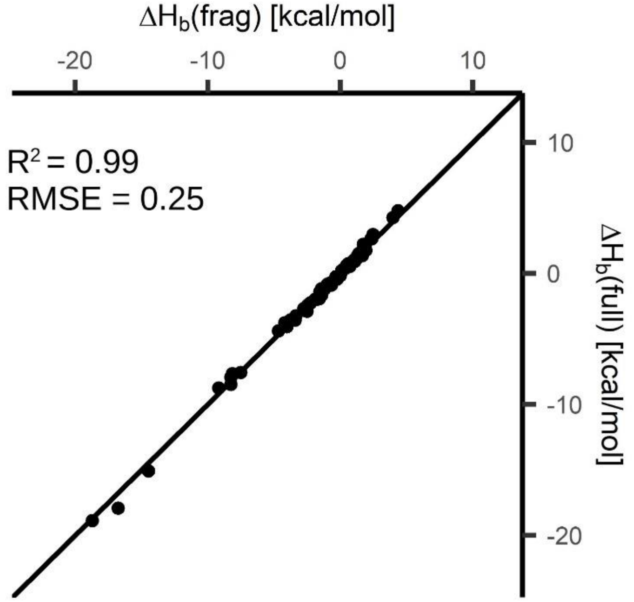

Figure S1 The effect of fragmentation on calculated ΔH$_b$ values. Correlation plot between calculated ΔH$_b$ values of individual water molecules obtained on fragmented and full structures of system 1lcj (78 data points). R^2 and RMSE (kcal/mol) values show an almost perfect correlation between respective values demonstrating that the effect of fragmentation on ΔH$_b$ is not significant.

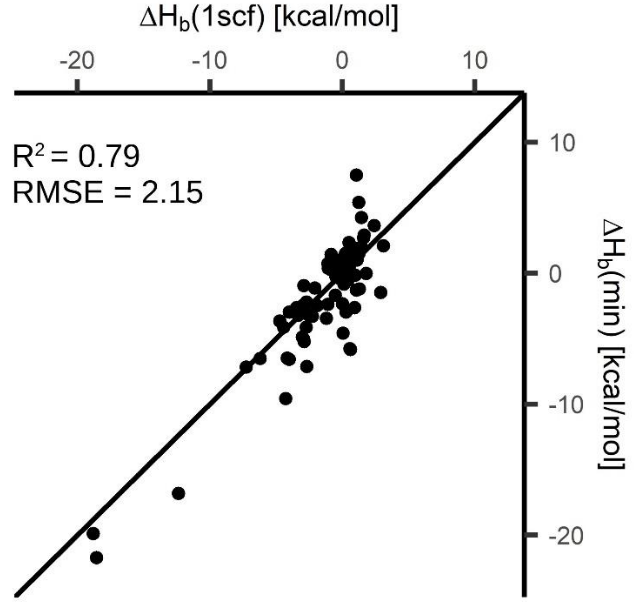

Figure S2 The effect of QM minimization on calculated ΔH$_b$ values. Correlation plot between calculated ΔH$_b$ values of individual water molecules of MM-minimized (with subsequent 1SCF) and QM-minimized (min) structures of system 1lcj (78 data points). R^2 and RMSE (kcal/mol) values show a strong correlation between single point and minimized ΔH$_b$ values.

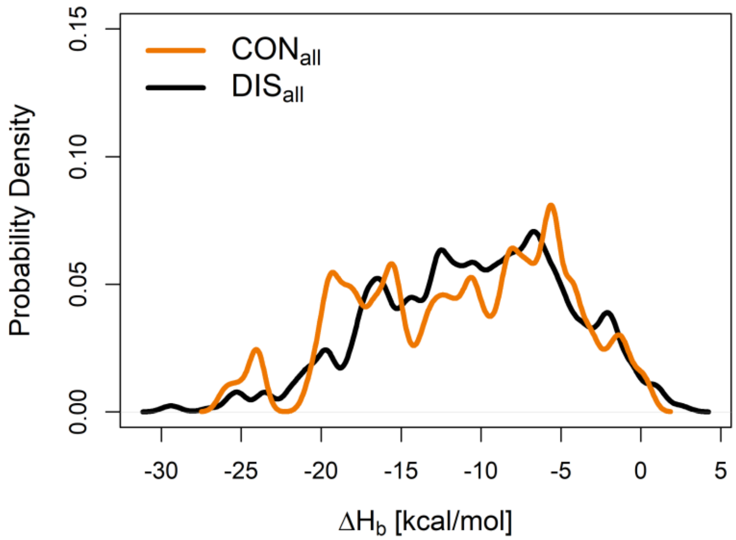

Figure S3 Distribution of conserved and displaced water sets with the inclusion of neighbouring (surrounding) water molecules in the ΔH_b calculation. Surrounding water molecules (sWAT) having a maximum distance of 3.5 Å from a specific water were added to the calculation as a part of the target (Target:sWAT, **Eq. S1**). Resulting distributions were fitted with the kernel density estimation technique and show a complete overlap of the conserved and displaced sets with no practical differences in local densities.

$$\text{Target:sWAT} + H_2O = \text{Target:sWAT:}H_2O$$

$$\Delta H_b = \Delta_f H(\text{Target:sWAT:}H_2O) - \Delta_f H(\text{Target:sWAT}) - \Delta_f H(H_2O) \quad \textbf{Eq. S1}$$

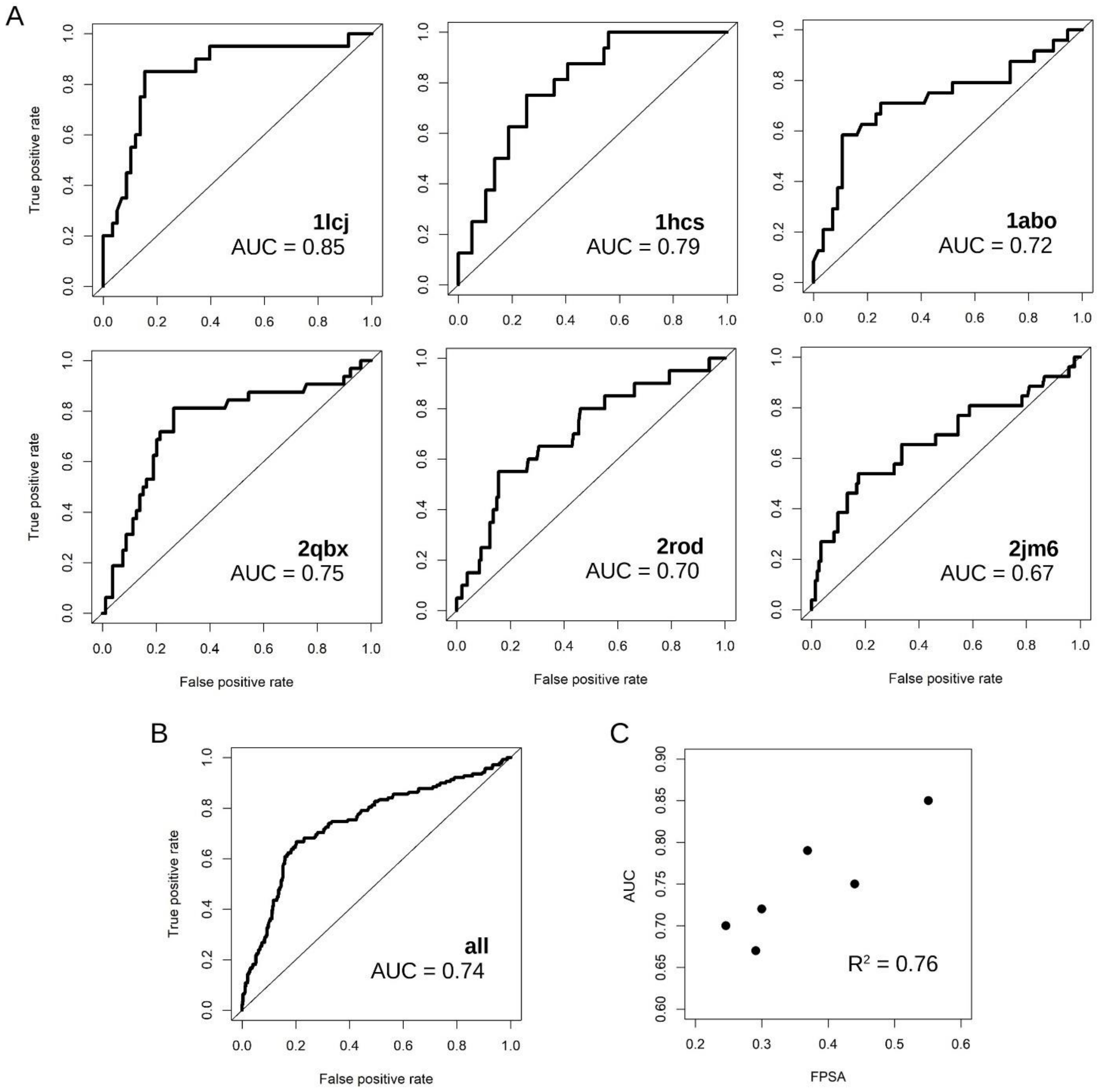

Figure S4 ΔHb-based classification efficiency of conserved and displaced water positions in terms of ROC analysis and its relation to binding site polarity. ROC AUC value of 0.74 obtained on the overall dataset (B) shows that water positions with larger negative ΔH_b are more likely to be conserved upon ligand binding. Notably, the ability of ΔH_b to discriminate between conserved and displaced water positions is somewhat varying for the investigated systems (A) and is more pronounced as the polarity of the binding pocket increases. This finding is supported by the strong correlation (R^2=0.76) between the Fractional Polar Surface Area of the binding pockets and the respective ROC AUC values (C). Details of the calculations are presented in **Table S9**.

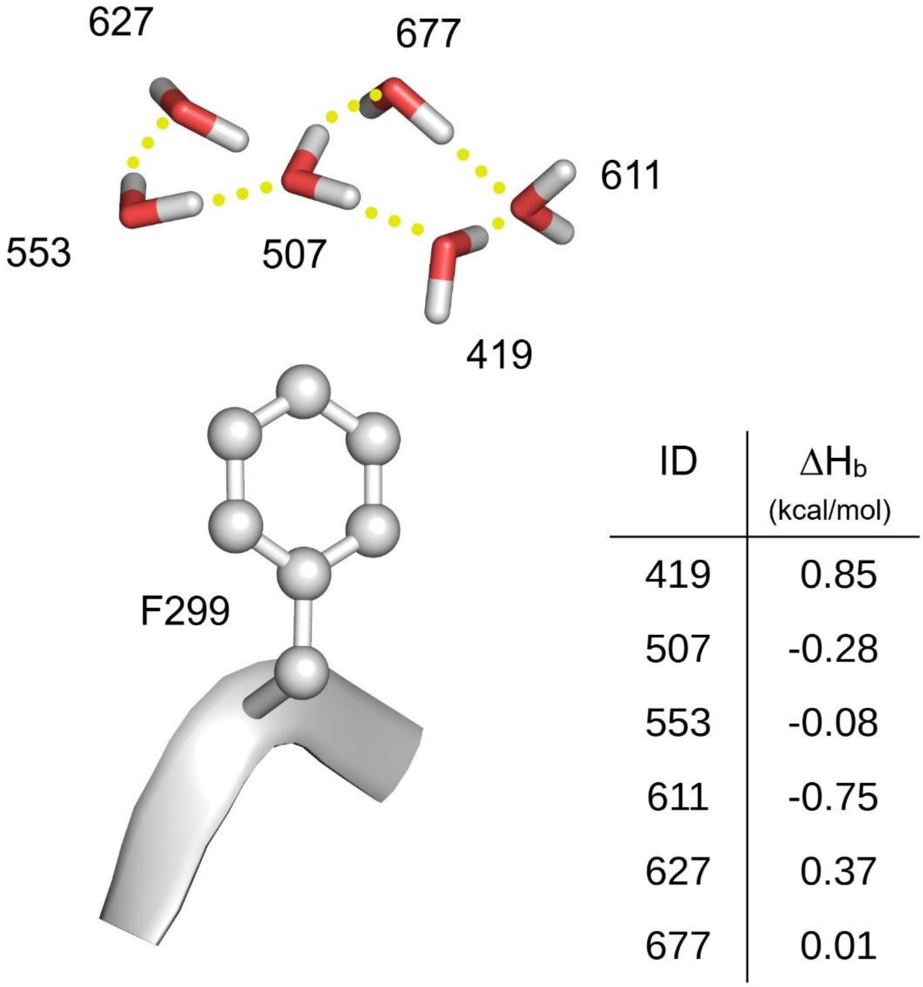

ID	ΔH_b (kcal/mol)
419	0.85
507	-0.28
553	-0.08
611	-0.75
627	0.37
677	0.01

Figure S5 Water structure in the proximity of non-polar target surface. The binding pocket of System 2jm6 is built up of mostly non-polar residues (multiple F, L, V and M residues, FPSA=0.291) inducing a hydrophobic environment for the ambient water molecules. As an example, in the neighbourhood of the F299 residue (ball stick, grey) waters (stick) tend to form hydrogen bonds (dashed dots, yellow) mostly with each other in the absence of H-bonding capabilities from the target side. The lack of interaction is apparent from the respective ΔH_b values that are centered around 0 kcal/mol. Such waters of unfavorable ΔH_b have high mobility and are easily displaceable by ligands.

Tables

Table S1 Methods for structural classification of binding site water molecules.

**Name**[a]	**Model**	**Descriptors**	$\mathbf{N_t}^b$	$\mathbf{N_w}^c$
Consolv[1]	k-nearest neighbours	• B-factor<br>• H-bond count<br>• Atomic density<br>• Atomic hydrophilicity	20	224
WaterScore[2]	logistic regression	• B-factor<br>• H-bond energy<br>• Protein atomic contact count<br>• Solvent-accessible surface-area	18	86
Ross et al.[3]	decision tree	• H-bond energy<br>• Hydrophilicity score<br>• Lipophilicity score	85	1004
Amadasi et al.[4]	nonlinear regression	• HINT score<br>• Rank score	22	193

[a]Name of the method or research group
[b]Number of distinct targets investigated
[c]Number of water molecules/hydration sites analyzed (training+validation)

Table S2 Methods for thermodynamic characterization of binding site water molecules.

Name[a]	Theory	Thermodynamic quantities	N_t[b]	N_w[c]
Double decoupling[5]	• Monte Carlo sampling<br>• Replica exchange themodynamic integration<br>• Bayesian probability model	ΔG	6	54
SPAM[6]	• MD sampling<br>• Partition function calculation based on water interaction energy distribution	ΔG, ΔE, TΔS	2	15
WATsite[7]	• MD sampling<br>• Interaction energy and translational-rotational distribution of hydration sites are evaluated	ΔG, ΔH, ΔS	3	136
WaterMap[8]	• MD sampling<br>• Inhomogenous fluid solvation theory	ΔG, ΔE, TΔS	4	75
SZMAP [9]	• Grid sampling<br>• Partition function calculation based on probe interaction energy distribution	ΔG, ΔH, TΔS	6	54
GCT[10]	• MD sampling<br>• Grid cell theory	ΔG, ΔH, ΔS	3	3

[a]Name of the method or group
[b]Number of distinct targets investigated
[c]Number of water molecules/hydration sites analyzed (training+validation)

Table S3 Target-ligand systems investigated in the present study.

PDB code	Target	Ligand	Resolution (Å)	#Waters[a] (apo)	#Waters[b] (holo)
**General classification and calibration**					
1abo	Abl tyrosine kinase SH3 domain	Ac-APTMPPPLPP-NH_2	2.2	80	59
1hcs	Human pp60c-src SH2 domain	Ac-pY-EEIE-NH_2	NMR	75	59
1lcj	P56-lck tyrosine kinase SH2 domain	Ac-EPQ-pY-EEIPIYL-NH_2	1.8	78	47
2jm6	Mieloid leukemia differentiation protein	PADLKDECAQLRRIGDKVNLRQKLLN	NMR	169	100
2qbx	Ephrine B receptor 4	SNEWIQPRLPQH	2.3	111	71
2rod	Mieloid leukemia differentiation protein	AELPPEFAAQLRKIGDKVYCTWSAPD	NMR	174	110
			**sum:**	**687**	**446**
**Case studies**					
1g6l	HIV-1 protease	see **Table S5**	1.9	38	-
6bkk	Influenza A M2 transmembrane domain	Amantadine	2.0	20	-

[a]Number of predicted water molecules in the binding pocket of the apo target (5Å cut-off, See Methods)

[b]Number of predicted water molecules in the target-ligand binding interface (5Å cut-off, See Methods)

Table S4 Counts of water molecules in different sets after structural classification (mtol = 1.0 Å).

System	Conserved (CON)	Displaced (DIS)	Sum
1abo	24	56	**80**
1hcs	16	59	**75**
1lcj	20	58	**78**
2jm6	26	143	**169**
2qbx	32	79	**111**
2rod	20	154	**174**
**All**	**138**	**549**	**687**

Table S5 HIV test set experimental data collection.

Inhibitor	Reference PDB (holo)	K_i (nM)	RMSD of fit to apo protein (Å)
**Amprenavir**	3nu3[11]	0.6[12]	0.7
**Atazanavir**	3eky[13]	2.66[14]	0.4
**Darunavir**	2ien[15]	1[15]	0.5
**DMP323**	3jvw[16]	0.31[17]	0.7
**Indinavir**	1hsg[18]	0.36[19]	0.5
**Lopinavir**	2q5k[20]	0.0013[21]	0.6
**Nelfinavir**	1ohr[22]	2[22]	0.6
**Ritonavir**	1hxw[23]	0.015[23]	0.6
**Saquinavir**	3oxc[24]	0.12[25]	0.5
**Tipranavir**	2o4p[26]	0.008[27]	0.5
**U89360E**	1gno[28]	20[29]	0.3

Table S6 Validation of predicted water positions. Crystallographic water positions of the used experimental structures with maximum B-factor values of 30 Å^2 in the binding site (maximum of 5 Å distance from the crystallographic ligand) were compared to predicted positions at a match tolerance of 1.5 Å. Success Rates (SR) are expressed as a percentage of matched crystallographic positions.

**System**	**N_{water}[a]**	**N_{match}[b]**	**SR[c] (%)**
1abo (holo)	6	5	83.3
1lcj (holo)	8	7	87.5
2qbx (holo)	4	4	100.0
1g6l (apo)	10	9	90.0
**Mean**			90.2

[a]Number of water molecules in the binding interface
[b]Number of matches between predicted and experimental water molecules
[c]Ratio of N_{match} to N_{water}

Table S7 Optimal ΔH_b classification thresholds (kcal/mol) and respective accuracy values based on the maximum F1-score statistic.

**System**	**Threshold (kcal/mol)**	**Accuracy (%)**
1abo	-2.0	80.0
1hcs	-1.4	74.7
1lcj	-1.9	84.6
2qbx	-2.1	75.7
2jm6	-2.1	78.1
2rod	-1.9	81.0
**All**	**-1.9**	**78.7**

Table S8 ROC AUC values of the ΔH_b-based classification of conserved water positions using single point QM calculation and QM minimization. The highly comparable ROC AUC values obtained on MM minimized and QM minimized structures imply that classification does not benefit from more precise water positions and orientations. QM minimization also has at least two orders of magnitude higher computational cost making it an impractical choice.

**System**	**Single point calculation**	**Minimization**
1abo	0.72	0.69
1hcs	0.79	0.77
1lcj	0.85	0.86
2qbx	0.75	0.72
2jm6	0.67	0.68
2rod	0.70	0.70
**All**	**0.74**	**0.73**

Table S9 Calculated Buried Surface Area (BSA), Buried Polar Surface Area (BPSA) and Fractional Polar Surface Area (FPSA) of the binding pockets of investigated systems. Calculations were performed with the COSMO module of Mopac. Atomic contributions of Solvent-Accessible Surface-Area (SASA)($Å^2$) were calculated for the target (apo) and target-ligand complex (holo) structures (keyword=COSWRT). The Buried Surface Area ($Å^2$) was estimated as the sum of the difference between the atomic SASA contributions of the target in the apo and holo structures. Buried Polar Surface Area ($Å^2$) was obtained as the sum of BSA contributions of O, N, S atoms and attached polar hydrogens. Fractional Polar Surface Area was defined as the ratio of BPSA to BSA.

System	BSA ($Å^2$)	BPSA ($Å^2$)	FPSA
1abo	342.0	102.6	0.30
1hcs	294.4	108.7	0.37
1lcj	318.9	175.7	0.55
2qbx	589.4	259.1	0.44
2jm6	791.9	230.3	0.29
2rod	758.6	186.2	0.25

Table S10 List of water molecules generated for the binding pocket of the HIV-1 protease target (PDB code 1g6l).

Notation[a]	ΔH_b [kcal/mol]	PDB residue number[b]
**W1**	**-14.41**	**1100**
**W2**	**-9.34**	**1178**
**W3**	**-7.78**	**1162**
**W4**	**-6.77**	**1124**
**W5**	**-6.44**	**1202**
**W6**	**-6.19**	**1358**
**W7**	**-6.13**	**1129**
**W8**	**-4.81**	**4695**
**W9**	**-4.72**	**2547**
**W10**	**-4.26**	**1103**
**W11**	**-3.99**	**1362**
**W12**	**-3.75**	**1219**
**W13**	**-3.65**	**1271**
**W14**	**-3.59**	**1122**
**W15**	**-3.54**	**1176**
**W16**	**-3.13**	**1254**
**W17**	**-2.71**	**1138**
**W18**	**-2.46**	**2055**
**W19**	**-2.43**	**1583**
**W20**	**-2.34**	**1245**
**W21**	**-2.26**	**1170**
**W22**	**-1.88**	**1411**
W23	-1.79	1399
W24	-1.56	1244
W25	-1.00	2290
W26	-0.55	1189
W27	-0.42	1226
W28	-0.42	1220
W29	0.30	2054
W30	0.33	1867
W31	1.04	1676
W32	1.37	1250
W33	2.19	1572
W34	3.12	1264
W35	3.34	1139
W36	3.58	2566
W37	4.54	1154
W38	6.03	1383

[a] Bold serial number indicate a water being classified into the CON_{1g6l} set.
[b] For the apo PDB file see Availability of data

Table S11 List of water molecules generated for the binding pocket of the influenza A ion channel target (PDB code 6bkk).

**Notation**[a]	**ΔH_b [kcal/mol]**	**PDB residue number**[b]
**W1**	**-11.20**	**66**
**W2**	**-10.42**	**55**
**W3**	**-10.09**	**69**
**W4**	**-6.56**	**74**
**W5**	**-5.59**	**78**
**W6**	**-5.06**	**73**
**W7**	**-4.29**	**67**
**W8**	**-3.27**	**70**
**W9**	**-1.93**	**84**
W10	-1.47	76
W11	-0.89	96
W12	-0.36	83
W13	0.53	116
W14	1.23	87
W15	2.04	86
W16	2.50	160
W17	2.57	820
W18	2.62	91
W19	2.90	199
W20	6.77	111

[a] Bold serial number indicate a water being classified into the CON_{6bkk} set.
[b] For the apo PDB file see Availability of data

References

(1) Raymer, M. L.; Sanschagrin, P. C.; Punch, W. F.; Venkataraman, S.; Goodman, E. D.; Kuhn, L. A. Predicting Conserved Water-Mediated and Polar Ligand Interactions in Proteins Using a K-Nearest-Neighbors Genetic algorithm11Edited by B. Honig. *Journal of Molecular Biology* **1997**, *265* (4), 445–464. https://doi.org/10.1006/jmbi.1996.0746.

(2) García-Sosa, A. T.; Mancera, R. L.; Dean, P. M. WaterScore: A Novel Method for Distinguishing between Bound and Displaceable Water Molecules in the Crystal Structure of the Binding Site of Protein-Ligand Complexes. *Journal of Molecular Modeling* **2003**, *9* (3), 172–182. https://doi.org/10.1007/s00894-003-0129-x.

(3) Ross, G. A.; Morris, G. M.; Biggin, P. C. Rapid and Accurate Prediction and Scoring of Water Molecules in Protein Binding Sites. *PLOS ONE* **2012**, *7* (3), e32036. https://doi.org/10.1371/journal.pone.0032036.

(4) Amadasi, A.; Surface, J. A.; Spyrakis, F.; Cozzini, P.; Mozzarelli, A.; Kellogg, G. E. Robust Classification of “Relevant” Water Molecules in Putative Protein Binding Sites. *J. Med. Chem.* **2008**, *51* (4), 1063–1067. https://doi.org/10.1021/jm701023h.

(5) Barillari, C.; Taylor, J.; Viner, R.; Essex, J. W. Classification of Water Molecules in Protein Binding Sites. *J. Am. Chem. Soc.* **2007**, *129* (9), 2577–2587. https://doi.org/10.1021/ja066980q.

(6) Cui, G.; Swails, J. M.; Manas, E. S. SPAM: A Simple Approach for Profiling Bound Water Molecules. *J. Chem. Theory Comput.* **2013**, *9* (12), 5539–5549. https://doi.org/10.1021/ct400711g.

(7) Hu, B.; Lill, M. A. WATsite: Hydration Site Prediction Program with PyMOL Interface. *Journal of Computational Chemistry* **2014**, *35* (16), 1255–1260. https://doi.org/10.1002/jcc.23616.

(8) Abel, R.; Young, T.; Farid, R.; Berne, B. J.; Friesner, R. A. Role of the Active-Site Solvent in the Thermodynamics of Factor Xa Ligand Binding. *J. Am. Chem. Soc.* **2008**, *130* (9), 2817–2831. https://doi.org/10.1021/ja0771033.

(9) Bayden, A. S.; Moustakas, D. T.; Joseph-McCarthy, D.; Lamb, M. L. Evaluating Free Energies of Binding and Conservation of Crystallographic Waters Using SZMAP. *J. Chem. Inf. Model.* **2015**, *55* (8), 1552–1565. https://doi.org/10.1021/ci500746d.

(10) Gerogiokas, G.; Southey, M. W. Y.; Mazanetz, M. P.; Hefeitz, A.; Bodkin, M.; Law, R. J.; Michel, J. Evaluation of Water Displacement Energetics in Protein Binding Sites with Grid Cell Theory. *Phys. Chem. Chem. Phys.* **2015**, *17* (13), 8416–8426. https://doi.org/10.1039/C4CP05572A.

(11) Shen, C.-H.; Wang, Y.-F.; Kovalevsky, A. Y.; Harrison, R. W.; Weber, I. T. Amprenavir Complexes with HIV-1 Protease and Its Drug-Resistant Mutants Altering Hydrophobic Clusters. *The FEBS Journal* **2010**, *277* (18), 3699–3714. https://doi.org/10.1111/j.1742-4658.2010.07771.x.

(12) Kim, E. E.; Baker, C. T.; Dwyer, M. D.; Murcko, M. A.; Rao, B. G.; Tung, R. D.; Navia, M. A. Crystal Structure of HIV-1 Protease in Complex with VX-478, a Potent and Orally Bioavailable Inhibitor of the Enzyme. *J. Am. Chem. Soc.* **1995**, *117* (3), 1181–1182. https://doi.org/10.1021/ja00108a056.

(13) King, N. M.; Prabu-Jeyabalan, M.; Bandaranayake, R. M.; Nalam, M. N. L.; Nalivaika, E. A.; Özen, A.; Haliloğlu, T.; Yılmaz, N. K.; Schiffer, C. A. Extreme Entropy–Enthalpy Compensation in a Drug-Resistant Variant of HIV-1 Protease. *ACS Chem. Biol.* **2012**, *7* (9), 1536–1546. https://doi.org/10.1021/cb300191k.

(14) Robinson Brett S.; Riccardi Keith A.; Gong Yi-fei; Guo Qi; Stock David A.; Blair Wade S.; Terry Brian J.; Deminie Carol A.; Djang Fred; Colonno Richard J.; Lin Pin-fang. BMS-232632, a Highly Potent Human Immunodeficiency Virus Protease Inhibitor That Can Be Used in Combination with Other Available Antiretroviral Agents. *Antimicrobial Agents and Chemotherapy* **2000**, *44* (8), 2093–2099. https://doi.org/10.1128/aac.44.8.2093-2099.2000.

(15) Tie, Y.; Boross, P. I.; Wang, Y.-F.; Gaddis, L.; Hussain, A. K.; Leshchenko, S.; Ghosh, A. K.; Louis, J. M.; Harrison, R. W.; Weber, I. T. High Resolution Crystal Structures of HIV-1 Protease with a Potent Non-Peptide Inhibitor (UIC-94017) Active Against Multi-Drug-Resistant Clinical Strains. *Journal of Molecular Biology* **2004**, *338* (2), 341–352. https://doi.org/10.1016/j.jmb.2004.02.052.

(16) Ishima, R.; Gong, Q.; Tie, Y.; Weber, I. T.; Louis, J. M. Highly Conserved Glycine 86 and Arginine 87 Residues Contribute Differently to the Structure and Activity of the Mature HIV-1 Protease. *Proteins: Structure, Function, and Bioinformatics* **2010**, *78* (4), 1015–1025. https://doi.org/10.1002/prot.22625.

(17) Rodgers, J. D.; Lam, P. Y. S.; Johnson, B. L.; Wang, H.; Ko, S. S.; Seitz, S. P.; Trainor, G. L.; Anderson, P. S.; Klabe, R. M.; Bacheler, L. T.; Cordova, B.; Garber, S.; Reid, C.; Wright, M. R.; Chang, C.-H.; Erickson-Viitanen, S. Design and Selection of DMP 850 and DMP 851: The next Generation of Cyclic Urea HIV Protease Inhibitors. *Chemistry & Biology* **1998**, *5* (10), 597–608. https://doi.org/10.1016/S1074-5521(98)90117-X.

(18) Chen, Z.; Li, Y.; Schock, H. B.; Hall, D.; Chen, E.; Kuo, L. C. Three-Dimensional Structure of a Mutant HIV-1 Protease Displaying Cross-Resistance to All Protease Inhibitors in Clinical Trials (∗). *Journal of Biological Chemistry* **1995**, *270* (37), 21433–21436. https://doi.org/10.1074/jbc.270.37.21433.

(19) Vacca, J. P.; Dorsey, B. D.; Schleif, W. A.; Levin, R. B.; McDaniel, S. L.; Darke, P. L.; Zugay, J.; Quintero, J. C.; Blahy, O. M.; Roth, E. L-735,524: An Orally Bioavailable Human Immunodeficiency Virus Type 1 Protease Inhibitor. *Proceedings of the National Academy of Sciences* **1994**, *91* (9), 4096–4100. https://doi.org/10.1073/pnas.91.9.4096.

(20) Reddy, G. S. K. K.; Ali, A.; Nalam, M. N. L.; Anjum, S. G.; Cao, H.; Nathans, R. S.; Schiffer, C. A.; Rana, T. M. Design and Synthesis of HIV-1 Protease Inhibitors Incorporating Oxazolidinones as P2/P2′ Ligands in Pseudosymmetric Dipeptide Isosteres. *J. Med. Chem.* **2007**, *50* (18), 4316–4328. https://doi.org/10.1021/jm070284z.

(21) Sham Hing L.; Kempf Dale J.; Molla Akhteruzammen; Marsh Kennan C.; Kumar Gondi N.; Chen Chih-Ming; Kati Warren; Stewart Kent; Lal Ritu; Hsu Ann; Betebenner David; Korneyeva Marina; Vasavanonda Sudthida; McDonald Edith; Saldivar Ayda; Wideburg Norm; Chen Xiaoqi; Niu Ping; Park Chang; Jayanti Venkata; Grabowski Brian; Granneman G. Richard; Sun Eugene; Japour Anthony J.; Leonard John M.; Plattner Jacob J.; Norbeck Daniel W. ABT-378, a Highly Potent Inhibitor of the Human Immunodeficiency Virus Protease. *Antimicrobial Agents and Chemotherapy* **1998**, *42* (12), 3218–3224. https://doi.org/10.1128/aac.42.12.3218.

(22) Kaldor, S. W.; Kalish, V. J.; Davies, J. F.; Shetty, B. V.; Fritz, J. E.; Appelt, K.; Burgess, J. A.; Campanale, K. M.; Chirgadze, N. Y.; Clawson, D. K.; Dressman, B. A.; Hatch, S. D.; Khalil, D. A.; Kosa, M. B.; Lubbehusen, P. P.; Muesing, M. A.; Patick, A. K.; Reich, S. H.; Su, K. S.; Tatlock, J. H. Viracept (Nelfinavir Mesylate, AG1343): A Potent, Orally Bioavailable Inhibitor of HIV-1 Protease. *J. Med. Chem.* **1997**, *40* (24), 3979–3985. https://doi.org/10.1021/jm9704098.

(23) Kempf, D. J.; Marsh, K. C.; Denissen, J. F.; McDonald, E.; Vasavanonda, S.; Flentge, C. A.; Green, B. E.; Fino, L.; Park, C. H.; Kong, X. P. ABT-538 Is a Potent Inhibitor of Human Immunodeficiency Virus Protease and Has High Oral Bioavailability in Humans. *Proceedings of the National Academy of Sciences* **1995**, *92* (7), 2484–2488. https://doi.org/10.1073/pnas.92.7.2484.
(24) Tie, Y.; Kovalevsky, A. Y.; Boross, P.; Wang, Y.-F.; Ghosh, A. K.; Tozser, J.; Harrison, R. W.; Weber, I. T. Atomic Resolution Crystal Structures of HIV-1 Protease and Mutants V82A and I84V with Saquinavir. *Proteins: Structure, Function, and Bioinformatics* **2007**, *67* (1), 232–242. https://doi.org/10.1002/prot.21304.
(25) Roberts, N. A.; Martin, J. A.; Kinchington, D.; Broadhurst, A. V.; Craig, J. C.; Duncan, I. B.; Galpin, S. A.; Handa, B. K.; Kay, J.; Kröhn, A.; Lambert, R. W.; Merrett, J. H.; Mills, J. S.; Parkes, K. E. B.; Redshaw, S.; Ritchie, A. J.; Taylor, D. L.; Thomas, G. J.; Machin, P. J. Rational Design of Peptide-Based HIV Proteinase Inhibitors. *Science* **1990**, *248* (4953), 358–361. https://doi.org/10.1126/science.2183354.
(26) Muzammil S.; Armstrong A. A.; Kang L. W.; Jakalian A.; Bonneau P. R.; Schmelmer V.; Amzel L. M.; Freire E. Unique Thermodynamic Response of Tipranavir to Human Immunodeficiency Virus Type 1 Protease Drug Resistance Mutations. *Journal of Virology* **2007**, *81* (10), 5144–5154. https://doi.org/10.1128/jvi.02706-06.
(27) Turner, S. R.; Strohbach, J. W.; Tommasi, R. A.; Aristoff, P. A.; Johnson, P. D.; Skulnick, H. I.; Dolak, L. A.; Seest, E. P.; Tomich, P. K.; Bohanon, M. J.; Horng, M.-M.; Lynn, J. C.; Chong, K.-T.; Hinshaw, R. R.; Watenpaugh, K. D.; Janakiraman, M. N.; Thaisrivongs, S. Tipranavir (PNU-140690): A Potent, Orally Bioavailable Nonpeptidic HIV Protease Inhibitor of the 5,6-Dihydro-4-Hydroxy-2-Pyrone Sulfonamide Class. *J. Med. Chem.* **1998**, *41* (18), 3467–3476. https://doi.org/10.1021/jm9802158.
(28) Hong, L.; Treharne, A.; Hartsuck, J. A.; Foundling, S.; Tang, J. Crystal Structures of Complexes of a Peptidic Inhibitor with Wild-Type and Two Mutant HIV-1 Proteases,. *Biochemistry* **1996**, *35* (33), 10627–10633. https://doi.org/10.1021/bi960481s.
(29) Lin, Y.; Lin, X.; Hong, L.; Foundling, S.; Heinrikson, R. L.; Thaisrivongs, S.; Leelamanit, W.; Raterman, D.; Shah, M. Effect of Point Mutations on the Kinetics and the Inhibition of Human Immunodeficiency Virus Type 1 Protease: Relationship to Drug Resistance. *Biochemistry* **1995**, *34* (4), 1143–1152. https://doi.org/10.1021/bi00004a007.
